# Exploring the five-paced viper (*Deinagkistrodon acutus*) venom proteome by integrating a combinatorial peptide ligand library approach with shotgun LC-MS/MS

**DOI:** 10.1590/1678-9199-JVATITD-2020-0196

**Published:** 2021-10-25

**Authors:** Xuekui Nie, Qiyi He, Bin Zhou, Dachun Huang, Junbo Chen, Qianzi Chen, Shuqing Yang, Xiaodong Yu

**Affiliations:** 1 Animal Toxin Group, Engineering Research Center of Active Substance and Biotechnology, Ministry of Education, College of Life Sciences, Chongqing Normal University, Chongqing, China.; 2 Library, Chongqing Normal University, Chongqing, China.; 3 Emergency Department, Chongqing Emergency Medical Center, Chongqing University Central Hospital, Chongqing, China.

**Keywords:** Combinatorial peptide ligand library, Deinagkistrodon acutus, Snake venom, Venomics, Trace toxins

## Abstract

**Background:**

Snake venoms are complex mixtures of toxic proteins or peptides encoded by various gene families that function synergistically to incapacitate prey. In the present study, in order to unravel the proteomic repertoire of *Deinagkistrodon acutus* venom, some trace abundance components were analyzed.

**Methods:**

Shotgun proteomic approach combined with shotgun nano-LC-ESI-MS/MS were employed to characterize the medically important *D. acutus* venom, after collected samples were enriched with the combinatorial peptide ligand library (CPLL).

**Results:**

This avenue helped us find some trace components, undetected before, in *D. acutus* venom. The results indicated that *D. acutus* venom comprised 84 distinct proteins from 10 toxin families and 12 other proteins. These results are more than twice the number of venom components obtained from previous studies, which were only 29 distinct proteins obtained through RP-HPLC for the venom of the same species. The present results indicated that in *D. acutus* venom, the most abundant components (66.9%) included metalloproteinases, serine proteinases, and C-type lectin proteins; the medium abundant components (13%) comprised phospholipases A_2_ (PLA_2_) and 5’-nucleotidases and nucleases; whereas least abundant components (6%) were aminopeptidases, L-amino acid oxidases (LAAO), neurotoxins and disintegrins; and the trace components. The last were undetected before the use of conventional shotgun proteomics combined with shotgun nano-LC-ESI-MS/MS, such as cysteine-rich secretory proteins Da-CRPa, phospholipases B-like 1, phospholipases B (PLB), nerve growth factors (NGF), glutaminyl-peptide cyclortransferases (QC), and vascular non-inflammatory molecules 2 (VNN2).

**Conclusion:**

These findings demonstrated that the CPLL enrichment method worked well in finding the trace toxin proteins in *D. acutus* venom, in contrast with the previous venomic characterization of *D. acutus* by conventional LC-MS/MS. In conclusion, this approach combined with the CPLL enrichment was effective for allowing us to explore the hidden *D. acutus* venomic profile and extended the list of potential venom toxins.

## Background

The five-paced viper, also known as the Chinese moccasin (*Deinagkistrodon acutus*, once named as *Agkistrodon acutus*), is a unique member of the monotypic genus *Deinagkistrodon* of the Viperidae family, reaching up to 2 m in length and weighing over 5 kg. It is a highly venomous snake in China and its venom’s LD_50_ ranges from 0.04 mg/kg to 10.0 mg/kg SC [1]. Most toxins in its venom are responsible for coagulopathy in the envenomation caused by *D. acutus* bites.

Undoubtedly, unraveling the biochemical composition of *D. acutus* venom is of great importance for understanding the molecular mechanism of envenoming led by its bites. Like other snake venoms, *D. acutus* venom is also a mixture of bioactive compounds, containing enzymes, non-enzyme proteins, and polypeptides with a wide variety of sequence motifs, which are essential for binding important physiological molecular targets with high affinity to kill or immobilize preys [2].

Before the emerging of proteomic and transcriptomic methodology and technology, most studies on the venom of *D. acutus* were focused on the individual components with biochemical characteristics, and several components have been described including metalloproteinases [3,4], phospholipases A_2_ [5,6], serine proteases [7,8], C-type lectin-like proteins [9], and other polypeptides with promising biological functions [9,10]. However, researches on a single component of snake venom do not completely reveal the clear composition of *D. acutus* venom.

To access the global profile of *D. acutus* venom at the molecular level, Qinghua et al. [2] adopted the strategy of large-scale expressed sequence tags (ESTs) sequencing from the venom gland of *D. acutus* cDNA library. They identified the transcripts that may represent a general panorama of the physiological events taking place in the venom glands, and surveyed gene expression from the very specialized secretory tissue, especially for those involved in coagulopathy [2]. In 2016, they sequenced the genome and transcriptome of the five-paced viper and conducted comparative genomic analyses[11]. The above-mentioned works will help solve the global composition of the five-paced viper venom.

With the advances of 2-DE, HPLC, and MS/MS techniques, the proteome of several snake venoms were profiled in the reported documents. Shotgun proteomic approach combined with LC-MS/MS is a viable, highly sensitive, and high-throughput technique that provides rapidly a global proﬁle of the protein and polypeptide components in a complex biological sample, such as protein mixture (snake venom) [12], cells [13], tissues, and organs [14]. 

Recently, to enrich the components with low abundance in biological samples, the combinatorial peptide ligand library (CPLL) method was used to explore the global protein profiles in many biological samples, including human urine [15] and serum [16], human platelets [17], human erythrocytes [18], chicken egg white [19] and yolk [20], and plant tissues [21]. The CPLL consists of a solid-phase combinatorial library of hexapeptides synthesized via a short spacer on poly (hydroxy methacrylate) beads according to the modified Merrifield approach [22], each bead displaying the same hexapeptides distributed throughout the core of the pearl. The ligand density can achieve a density of ca. 40-60 mmol/mL bead volume, and the complete library may contain a set of millions of different hexapeptides.

CPLL beads reduce the dynamic range of the proteome by simultaneously diluting high-abundance proteins and concentrating low-abundance ones. Especially in the field of animal venomics, CPLL was used to unravel the global protein profiles of venoms in several venomous animals, such as honeybee (*Apis mellifera*) [23], *Crotalus atrox* [24] that led to the discovery of some trace protein components previously undetected. 

In the current work, we firstly explored an in-depth analysis of venomic of *D. acutus* by CPLL combined with shotgun nano-LC-ESI/MS/MS, which revealed a clear feature of protein distribution in *D. acutus* venom, which will facilitate to understand the mechanism of envenoming caused by *D. acutus* bites.

## Methods

### Venom collection

The adult snakes (*D. acutus*) with an average length of about 60 cm were captured in Wulingshan Mountain, Chongqing, China. The snake venoms were milked and lyophilized, then stored at −80 °C until further use.

### Protein enrichment by CPLL

Two hundred mg of lyophilized venom was dissolved in 3 mL of Tris-HCl buffer (pH 7.2, including 50 mM KCl) at room temperature overnight. The sample was centrifuged at 13, 000 × g for 10 min, then the supernatant was incubated with 100 µL of ProteoMiner™ beads (Catalog # 163-3009, Bio-Rad Laboratories, Inc, USA) pre-equilibrated with the same buffers, shaking for 3 h at room temperature. Subsequently, the non-specific absorbed proteins were removed by 3 rounds of washing using 10 mM NaH_2_PO_4_ buffer (pH 7.4, including 150 mM NaCl), finally, the captured proteins left on beads were eluted out using 200 µL, 100 µL, and 100 µL elution reagent (8 M urea, 2% CHAPS), respectively, and all the eluates were pooled to a protein concentration, as determined by Bradford protein assay (Thermo Scientific Pierce, Hudson, NH, USA).

### SDS-PAGE

The lyophilized venom and the eluate fraction with the same amount (approximately 40 μg) were loaded on 12% Tris-glycine SDS-PAGE in a Mini protean 3 system (Bio-Rad Laboratories, Hercules, CA, USA), running at 140 V. After electrophoresis, the gel was stained with colloidal coomassie blue.

### In-solution digestion

Protein digestion was performed according to the FASP procedure described by Wiśniewski et al. [25]. Briefly, the protein sample (about 30 μg) was solubilized in 30 μL SDT buffer (4% SDS, 100 mM DTT, 150 mM Tris-HCl pH 8.0) at 90°C for 5 min. The detergent, DTT, and other low-molecular-mass components were removed using 200 μL UA buffer (8 M Urea, 150 mM Tris-HCl pH 8.0) by repeated ultrafiltration (Microcon units, 3 kDa). Then 100 μL 0.05 M iodoacetamide in UA buffer was added to block reduced cysteine residues and the samples were incubated for 20 min in darkness. The filter was washed with 100 μL UA buffer three times and then with 100 μL 25 mM NH_4_HCO_3_ twice. Finally, the protein suspension was digested with 2 μg trypsin (Promega) in 40 μL 25 mM NH_4_HCO_3_ overnight at 37°C, and the resulting peptides were collected as a filtrate. 

### LC-ESI-MS/MS

Experiments were performed on a Q Exactive mass spectrometer that was coupled to Easy nano-LC (Proxeon Biosystems, now Thermo Fisher Scientific). A label-free quantification method (Q exactive) was used to achieve relative quantification by comparing the intensity of mass spectral peaks of different peptides. Six microliters of each fraction was injected for nano-LC-ESI-MS/MS analysis. The peptide mixture (5 μg) was loaded onto the C18 reversed-phase column (Thermo Scientific Easy Column, 10 cm long, 75 μm inner diameter, 3μm resin) in buffer A (0.1% formic acid) and separated with a linear gradient of buffer B (80% acetonitrile and 0.1% formic acid) at a flow rate of 250 nL/min controlled by IntelliFlow technology over 140 mins. MS data were acquired using a data-dependent top10 method dynamically choosing the most abundant precursor ions from the survey scan (300-1800 m/z) for HCD fragmentation. The determination of the target value is based on predictive Automatic Gain Control (pAGC). The dynamic exclusion duration was 60 s. Survey scans were acquired at a resolution of 70,000 at m/z 200 and resolution for HCD spectra was set to 17,500 at m/z 200. The normalized collision energy was 30 eV and the underfill ratio, which specifies the minimum percentage of the target value likely to be reached at maximum fill time, was defined as 0.1%. The instrument was run with peptide recognition mode enabled. 

### Sequence database searching and data analysis

MS/MS spectra were interpreted by using the Mascot search engine (Matrix Science, London, UK; version 2.2). The proposed peptide sequences were compared with nonredundant databases of snake venomous proteins generated from data compiled at the UniProtKB/Swiss-Prot and UniProtKB/TrEMBL (score ≥ 20). For protein identification, the following options were used. Peptide mass tolerance = 20 ppm, MS/MS tolerance = 0.1 Da, Enzyme = Trypsin, Missed cleavage = 2, Fixed modification: Carbamidomethyl (C), Variable modification；Oxidation(M). The high-confidence proteins were determined by the standard of unique peptide count ≥ 2.

## Results

### Protein compounds of venom treated with CPLL

**Figure 1.**
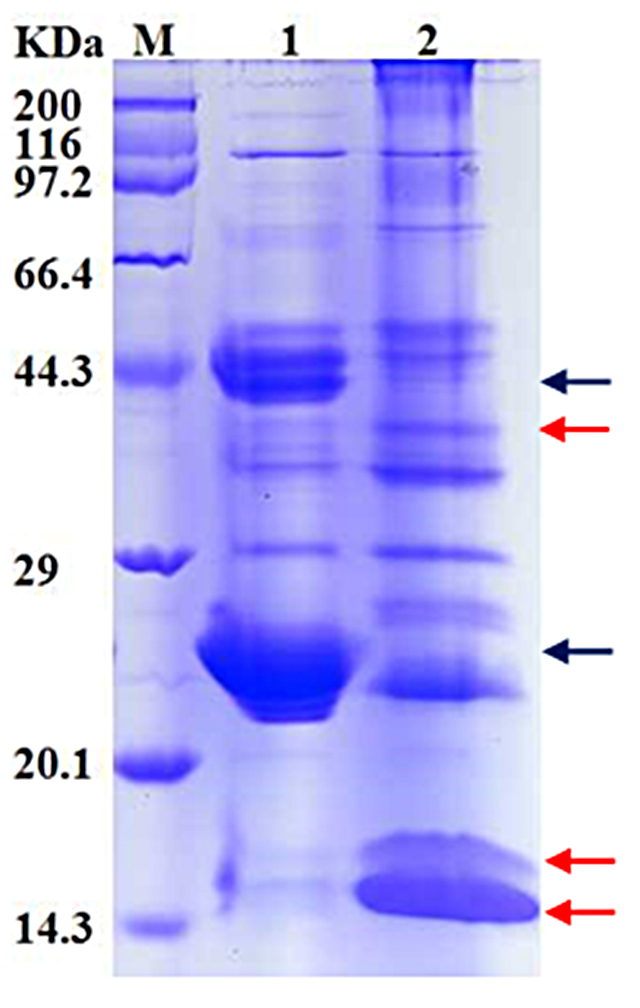
Electrophoretic separation of a combinatorial peptide ligand library (CPLL)-treated *D. acutus* venom sample. Crude *D. acutus* venom proteins (lane 1) and the elution fractions from CPLL (lane 2) were separated on a 12% reduced Tris-glycine SDS-PAGE gel. Markers were a mass ladder of pure proteins from 6.5 to 200 kDa. All samples were loaded in 10 µL volume (40 µg of protein), staining with colloidal coomassie blue. Red arrows presented the concentrated components, black arrows presented the diminished components.

The CPLL pretreatment decreased the dynamic range of the venom sample (Figure 1). The two most abundant proteins, such as snake venom metalloproteinase (SVMP) and serine protease (SVSP), were diminished in the elution fraction compared to the crude venom sample. From the SDS-PAGE pattern, we observed an enrichment protein band at around 17 kDa, which might contain a large number of 17 kDa protein family members, like C-type lectin. Besides, the three novel protein bands were found in the elution fraction. Further highlighting the usefulness of the combinatorial peptide library approach as a complementary tool of classical protocols is essential for lessening the high abundant compounds and concentrating the low-abundance and trace proteins from the complex venom proteomes.

### Identification of *D. acutus* venom proteins

*D. acutus* venom proteins were dealt with CPLL followed by trypsin digestion directly, then the tryptic peptide fractions were analyzed using the nano-LC-ESI-MS/MS proteomics technique. A total of 583 unique tryptic proteins or peptides were identified from *D. acutus* venom samples (Additional file 1). Eighty-four unique proteins were high confidence determined by more than two unique peptides matched. The information regarding the identified proteins, such as the matched peptide sequences, accession number, theoretical pI, and molecular mass, their charge states, and M/Z values, are given in (Additional file 1). This list includes 33 compounds found in venom protein and 44 compounds found in venom gland transcripts (Additional file 2). Besides, this study detected 47 new venom compounds in *D. acutus* venom, undetected before, which were also found in other species. 

### Characterization of venom protein profile

**Figure 2.**
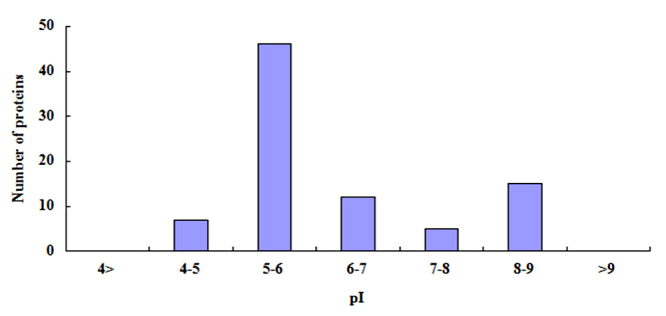
Distributions of isoelectric point (pI) of the high-confidence *D. acutus* venom proteins from the elution fraction from CPLL.

**Figure 3.**
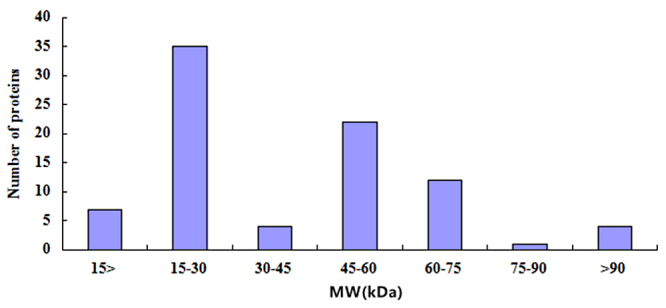
Distributions of the molecular mass of the high-confidence *D. acutus* venom proteins from the elution fraction from CPLL.

According to predicted amino acid sequences, the theoretical isoelectric point (pI) and molecular mass of the identified proteins were calculated using the computer pI/ molecular mass tool (http://cn.expasy.org/tools/pi_tool.html). There was an overview of the distribution of proteome components (Figure 2 and Figure 3) and showed that 54% (46/85) of the total proteins were distributed in a range of pI 5-6, but proteins with pI > 9 or pI < 4 were never found. The molecular mass of the identified proteins was mainly distributed in 15 kDa - 30 kDa (41.17%), 45 kDa-60 kDa (25.88%), and 60 kDa-75 kDa (14.12%). Especially, the proteins from 15 kDa to 75 kDa were accounted for 85.88% (73/85), the proteins with greater than 90 kDa accounted for 4.7% (4/85) and the proteins with lesser than 15 kDa accounted for 8.24% (7/85). 

### Overall protein composition of *D. acutus* venom

**Figure 4.**
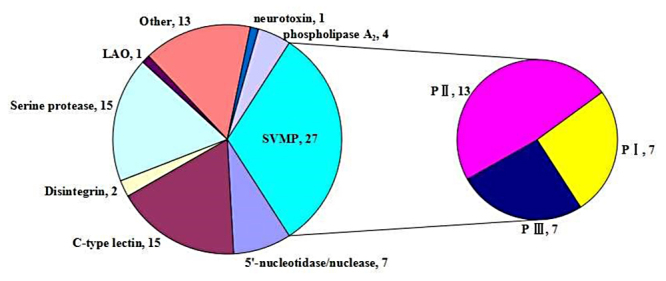
Overall protein composition of *D. acutus* venom. The relative number of different toxin families in all high-confidence proteins. P I, P II, P III -SVMP, snake venom metalloproteinase from class I, II, and III; C-type lectin, C-type lectin-like protein; LAAO, L-amino acid oxidase.

Identified *D. acutus* venom proteins belong to 10 groups of toxins (Figure 4, Table 1). The dominant protein families SVMPs (31.7%), SVSPs (17.6%), and C-type lectins (17.6%), the medium-abundance families PLA_2_ (4.7%), 5'-nucleotidase (5.9%), and nuclease (2.4%), and the low-abundance protein family aminopeptidase (1.2%), LAAO (1.2%), neurotoxin (1.2%), disintegrin (2.4%) amounting to 6% of the venom proteins. In the previous study, the venomic profile of *D. acutus* was composed of SVMPs (46.86%), C-type lectins (37.59%), PLA_2_ (7.33%), SVSP (6.62%), others (1.6%) [26]. Furthermore, the trace proteins, undetected before by conventional shotgun proteomics combined with LC-MS/MS, such as cysteine-rich secretory protein Da-CRPa, phospholipase B-like 1, PLB, NGF, glutaminyl-peptide cyclortransferase (QC), and vascular non-inflammatory molecule 2. 

**Table 1.** Overview of the relative occurrence of proteins (in the percentage of all high-confidence *D. acutus* venom proteins from the elution fractions from CPLL) of the different families in the venom of *D. acutus*.

Protein family	% of total venom proteins
P I-SVMP	8.2
P II-SVMP	15.3
P III-SVMP	8.2
C-type lectin	17.6
SVSP	17.6
5'-nucleotidase	5.9
PLA_2_	4.7
nuclease	2.4
Disintegrin	2.4
Aminopeptidase	1.2
LAAO	1.2
Neurotoxin	1.2
Other proteins	14.1

## Discussion

To further detect the low-abundance or trace components in *D. acutus* venom, we employed CPLL beads to treat the venom sample. Indeed, the *D. acutus* venom sample treated by CPLL beads displayed much more different molecular mass protein bands on Tris-glycine SDS-PAGE than the venom sample lack CPLL beads pretreatment (Figure 2), and the dominant components SVMP and serine protease were diminished in the venom [27], and the low-abundance components concentrated obviously. Thus, the CPLL pretreatment decreased the dynamic range of the *D. acutus* venom sample, extended its search towards the complete molecular mass range of the venom, and is an effective enrichment approach for detecting the low-abundance or trace components of venoms, as demonstrated in several previous reports, such as honeybee [23], Western diamondback rattlesnake [24] and African puff adder [22].

The CPLL couple to shotgun nano-LC-ESI-MS/MS allows us to explore *D. acutus* venomic. In total, 84 compounds were detected, which were categorized into 10 groups of toxins according to their putative function (Additional file 2). Among these, 32 compounds were found in venom protein and 44 compounds found in venom gland transcripts. The previous study about *D. acutus* venomic that 29 unique proteins or peptides had been identified using the shotgun digestion approach alone [26]. In our result, the protein family of the snake venom proteome and the members of the protein family were richer than previous reports [28], and we found that some dominant components (SVMP, C-type lectins, PLA_2_) were diminished and some component rich (SVSP) against previous studies. The most obvious variance between the previous and the present study was whether minor or trace components were discovered in the snake venom. We confirmed that our proposal was extremely valid. Meanwhile, the content of the main components showed significant differences, which may account for individual, geographical (habitats in Chongqing and Taiwan), age-related differences, CPLL [29-31]. Their biological function provides insights into venom toxicity. In snake venoms, components that are usually abundant are also functionally abundant, evolve more rapidly, and act on multiple targets [32]. Besides, snake injuries are often induced by the synergistic action of multiple snake venom components [33]. Our findings underscore the usefulness of combinatorial peptide libraries and shotgun nano-LC-ESI-MS/MS as powerful tools for visualization of venom proteomes. Through the combination of peptide library and mass spectrometry, a more comprehensive snake venomic was obtained, which helped to understand the molecular mechanism of *D. acutus* bites and guide the rational use and preparation of antivenom for snakebite.

As most viper snake venoms profile, *D. acutus* venom is mainly composed of SVMP, SVSP, C-type lectins, 5’-Nucleotidase, nuclease, PLA_2_ [34]. The pathological symptoms of *D. acutus* envenomation showed severe coagulopathy and thrombocytopenia in addition to localized ulceration and systemic bleeding. However, these pathological symptoms are mainly related to SVMPs in *D. acutus* venom, which displays a wide range of biological activities affecting hemostasis, including hemorrhagic, fibrinolytic, activation of prothrombin, and Factor X31-33 [35]. The previous report confirmed that post-translational mechanisms and transcript alternative splicing can expand the diversity of functions and structure of SVMP [36]. Excluding SVMP, some other toxins and non-toxins components also contribute to snakebite damage. Fifteen serine proteases were found in the *D. acutus* venom, and 6 SVSPs had never been reported in *D. acutus*. Indeed, 13 types of serine protease were found in the transcripts of the *D. acutus* venom gland, the results indicate that the mRNAs experienced the post-transcriptional processing (alternative mRNA splicing) that guided the synthesis of SVSPs in snake venom. Alternative mRNA splicing was involved in the production of divergent gene transcripts in some other species, such as Habu Snake, Protobothrops flavoviridis [36]. Fifteen C-type lectins were found and 3 C-type lectins had never been reported in *D. acutus*, surprisingly, 28 types of C-type lectin are found in the venom gland transcripts of *D. acutus*. C-type lectin affects thrombosis and hemostasis, and serine proteinases interfere in platelet aggregation, blood coagulation, and fibrinolysis [37]. The diverse and abundant snake venom families always play an indispensable role in snakebite morbidity and mortality.

The minor components are composed of disintegrin, PLB, phosphodiesterase (PDE), LAAO, NGF, aminopeptidase, glutaminyl-peptide cyclotransferase (QC), VNN2, cysteine-rich secretory protein Da-CRPa, and neurotoxin. Disintegrins act as receptor antagonists, inhibiting platelet aggregation induced by ADP, thrombin, and collagen [38]. In our study, only 2 disintegrins were found, not including those derived from metalloproteinase. Noticeably, most of the disintegrins derived from metalloproteinase with disintegrin-like domains, especially P-II class metalloproteinase (metalloproteinase-disintegrin precursor) cleaved by protease (proteolytic enzyme in venom), illuminating the diversity of metalloproteinase (P-I) and disintegrin [39]. Hence, plentiful disintegrins might exist in snake venom. PLB can cleave ester linkages at the positions sn-1 and sn-2 of glycerophospholipids from cell membranes [39], and hydrolyze the second messengers cAMP and cGMP, to regulate their intracellular concentration and biological effects [40]. 

Previous reports indicate that PDE plays a role in envenomation by hydrolyzing DNA and RNA, releasing adenosine and other purine nucleotides [41], and induces a variety of pathological and pharmacological effects, such as vascular permeability, hypotension, platelet aggregation, edema, and paralysis [42]. LAAO (ACTX-6), isolated from *D. acutus* venom, showed substrate specificity, cytotoxicity, antitumor activity in vivo, and apoptosis-inducing activity [43]. As for the antioxidant and antibacterial activities of LAAO, maybe as a self-protection mechanism to avoid diseases caused by the pathogenic bacteria due to the feeding process [44]. Aminopeptidase contributes a significant role in kallikrein-kinin system (angiotensin III, angiotensin II) and causes hypotension [45,46], while the capacity of aminopeptidase N and aminopeptidase A are diverse in peripheral and central of humans. The fundamental role of leucine aminopeptidase (arylamidase) contains digestion, hypotension, and anticoagulation [47]. 

QC, containing a catalytically essential zinc ion, catalyzes the conversion of protein N-terminal glutamine (or glutamic acid) residue into pGlu (pyroglutamate) and that is important during the maturation of many bioactive peptides, hormones, and proteins in their secretory pathway and also committed in cancer immunotherapy [48,49]. QC isolated from several animal and plant sources [50,51], and the genes coding for QC identified in numerous organisms [52,53], 8 snake venom QC protein sequences had been sequenced. 

Mature PIII-SVMPs secreted into the venom proteome usually contain an N-terminal pyroglutaminyl residue, suggesting that glutamyl cyclase plays an important role in the transformation of metalloproteinases precursors become mature metalloproteinases. VNN2 protein belongs to a novel glycosylphosphatidyl-inositol‑anchored protein member of the VNN family (belongs to a wider pantetheinase family) that serves an important role in transendothelial migration of cells and participates in regulating neutrophil trafficking and adherence [54,55], also serve a role in redox regulation, which may be associated with tumor progression in vitro [56]. Therefore, the cancer suppression mechanism that includes VNN2 is not only limited to mammals, and the same mechanism may exist in the serpent. Using proteomic and transcriptomic approaches, VNN2 was identified in king cobra [57], and we hypothesize that VNN2 (V8N7Y3) was responsible for the post-translational modification (ubiquitination) of the snake venom proteome. 

Unquestionably, snakebite envenomation syndrome attributes to the synergy of multiple toxic proteins or peptides. The minor abundant snake venom toxins activity to the wound adjacent target cell molecular, such as DNA, RNA, second messenger (cAMP, cGMP), so we confirm that secondary damages of snakebite may owe to the low abundant toxins [58]. Hence, we should not only focus on high-abundance components in snakebite treatment procedures, but trace components also should be taken seriously.

Snake venom is a treasure of potential drugs of protein or peptide origin. Snake venom-derived drugs currently in use or clinical trials are mainly used for the treatment of cardiovascular diseases. The non-addictive analgesic cobrotoxin from *Naja atra*; Captopril, an anti-hypertensive drug from *Bothrops jararaca*; Hemocoagulase from *B. atrox* for orthopedic surgery, abdominal surgery, and human vitrectomy; and a variety of drugs in clinical trials for acute peripheral arterial Alfimeprase from *Agkistrodon contortrix*, and *A. rhodostoma* for acute ischemic stroke [59]. And a variety of drugs have been isolated and purified from *A. contortrix*, including thrombolytic proteins [60], anticancer proteins [61]. With the application of “omics” technology in the process of snake venom research, we believe that a large number of snake venom proteins with potential drug value will be discovered one after another, and open up channels for the development of protein-based drug resources.

## Conclusion

Snake venom is a cocktail of proteins and peptides and each component plays an indispensable role in predation and defense. Numerous studies are committed to figuring out the venom profile, exploiting the “traditional method”, which did not detect the hidden or trace components in snake venom. The “omics method” comprises an intelligent alternative. The combination of the CPLL approach with nano-LC-ESI-MS/MS allows us to explore *D. acutus* venomics. In total, 84 compounds were detected, which were categorized into 10 different groups of toxins according to their putative function. Among these, 32 compounds were found from venom and 44 compounds were identified from venom gland transcripts. Their biological function provides insights into venom toxicity. The results underscore the usefulness of combinatorial peptide libraries and shotgun nano-LC-ESI-MS/MS as a powerful tool for visualization of venom proteome. Moreover, such combined method represents a powerful tool for tracing hidden components, and provides a guide for snakebite immunotherapy.

### Abbreviations

CPLL: combinatorial peptide ligand library; ESTs: expressed sequence tags; LAAO: L-amino acid oxidase; NCBI: National Center for Biotechnology Information; NGF: nerve growth factor; PDE: phosphodiesterase; pI: isoelectric point; PLA_2_: phospholipases A_2_; PLB: phospholipase B; QC: glutaminyl-peptide cyclotransferase; SVMP: snake venom metalloproteinase; SVSP: snake venom serine protease; VNN2: vascular noninflammatory molecule 2.

## Supplementary material

The following online material is available for this article:

Additional file 1. The information of protein identification from *D. acutus* venom determined by LC-MS/MS.

Additional file 2. Proteins identified from *D. acutus* venom.
