# Supplementary material for: Exploring the five-paced viper (Deinagkistrodon acutus) venom proteome by integrating a combinatorial peptide ligand library approach with shotgun LC-MS/MS
Source: J Venom Anim Toxins Incl Trop Dis. 2021 Oct 25;27:e20200196. doi: 10.1590/1678-9199-JVATITD-2020-0196 (PMC8547348; doi:10.1590/1678-9199-JVATITD-2020-0196)
Supplement: Additional file 1. [file 1678-9199-jvatitd-27-e20200196-s1.pdf]

J Venom Anim Toxins incl Trop Dis
https://doi.org/10.1590/1678-9199-JVATITD-2020-0196

Supplementary Material to "Exploring the five-paced viper (*Deinagkistrodon acutus*) venom proteome by integrating a combinatorial peptide ligand library approach with shotgun LC-MS/MS"

Additional file 1. The information of protein identification from *D. acutus* venom determined by LC-MS/MS.

Protein no.	Peptide mass/ charge	Peptide sequence	Sequence coverage (%)	Protein name	Acc. no.	Theor. mass /theor. PI
1	653.37/2+	DIIHR	32.2	Snake venom metalloproteinase AaPA	Q2E126	46757/5.3
	1546.8/2+	DLINVETSAENTLK				
	1450.78/3+	ENPPCILNKPLR				
	1539.8/3+	RSVGVVQDHSSVNR				
	781.36/2+	SFGEWR				
	1498.61/2+	YFSDCSYIQCR				
	798.33/2+	YNGDSDK				
	852.5/2+	AKDLIHR				
	639.33/2+	EYISK				
	2903.23/3+	HDEGSCSCGSGYTCIM*SPVINSEVIK				
	2887.24/3+	HDEGSCSCGSGYTCIMSPVINSEVIK				
	3337.57/3+	ISHDNAQLLTATDFDGPTIGLAYVASM*CDPK				
	3321.58/3+	ISHDNAQLLTATDFDGPTIGLAYVASMCDPK				

Protein no.	Peptide mass/ charge	Peptide sequence	Sequence coverage (%)	Protein name	Acc. no.	Theor. mass /theor. PI
	1383.7/3+	SVGVVQDHSSVNR				
2	1877.87/2+	AWVYEM*INTISESYR	33.9	Acutusin 1	D2Y163	46675/5.46
	1861.88/2+	AWVYEMINTISESYR				
	653.37/2+	DIIHR				
	1546.8/2+	DLINVETSAENTLK				
	1450.78/3+	ENPPCILNKPLR				
	781.36/2+	SFGEWR				
	1498.61/2+	YFSDCSYIQCR				
	812.38/2+	YKGDSDK				
	852.5/2+	AKDLIHR				
	639.33/2+	EYISK				
	3337.57/3+	ISHDNAQLLTATDFDGPTIGLAYVASM*CDPK				
	3321.58/3+	ISHDNAQLLTATDFDGPTIGLAYVASMCDPK				
	2089.06/2+	YLYIDIIVSALEM*WSEK				
	2073.06/2+	YLYIDIIVSALEMWSEK				
	1859.89/3+	YM*EIVIVVDHSM*YTK				
	1843.9/2+	YM*EIVIVVDHSMYTK				
	1827.9/3+	YMEIVIVVDHSMYTK				
3	1410.57/2+	CNCNVCIM*APR	25.66	Snake venom metalloproteinase aculysin-1	Q9W7S2	47304/5.89

Protein no.	Peptide mass/ charge	Peptide sequence	Sequence coverage (%)	Protein name	Acc. no.	Theor. mass /theor. PI
	1394.58/ 2+	CNCNVCIMAPR				
	688.42/2 +	DILTVK				
	2378.23/ 3+	EIFKPLNLDITLSGIEM*WDK				
	2506.33/ 3+	EIFKPLNLDITLSGIEM*WDKK				
	2362.24/ 2+	EIFKPLNLDITLSGIEMWDK				
	2490.33/ 3+	EIFKPLNLDITLSGIEMWDKK				
	976.58/2 +	FLTDKKPK				
	820.44/2 +	TAATDTLK				
	1478.59/ 2+	TFSDCSNNDYQK				
	2581.24/ 2+	IDNDNAQLQTAVDFDGETVGLAFK				
	773.42/2 +	LNTNPSK				
	646.35/2 +	TIQER				
	604.35/2 +	VNIMK				
4	688.42/2 +	DILTVK	33.66	Snake venom metalloproteinase H5	Q9IA Y2	46033/5. 75
	976.58/2 +	FLTDKKPK				
	2012.06/ 2+	QQTWPQTSVNLQLVVDR				
	820.44/2 +	TAATDTLK				
	1478.59/ 2+	TFSDCSNNDYQK				
	842.35/2 +	YNSDSEK				

Protein no.	Peptide mass/ charge	Peptide sequence	Sequence coverage (%)	Protein name	Acc. no.	Theor. mass /theor. PI
	2581.24/ 2+	IDNDNAQLQTAVDFDGETVGLAFK				
	773.42/2 +	LNTNPSK				
	599.29/2 +	SMYAK				
	604.35/2 +	VNIMK				
	4064.93/ 5+	YSAGIIQDHSAIPLLMAVTMAHELGHN LGMDHDDTYK				
5	1499.76/ 3+	HDNAQLITGIDFR	37.77	Recombinant fibrinogenase II	A2TK 72	26712/5. 14
	2138.09/ 3+	ITVKPDVDYTLNAFAEWR				
	1627.86/ 3+	KHDNAQLITGIDFR				
	1684.64/ 2+	M*YEAANNM*NEM*YR				
	1668.65/ 2+	M*YEAANNM*NEMYR				
	1652.65/ 2+	M*YEAANNMNEMYR				
	1668.65/ 2+	MYEAANNM*NEM*YR				
	1652.65/ 2+	MYEAANNM*NEMYR				
	1636.66/ 2+	MYEAANNMNEMYR				
	837.47/2 +	TYLLAEK				
	824.38/2 +	YNGDLDK				
	1065.56/ 2+	YNGDLDKIK				
	1198.64/ 2+	YVETVFVVDK				
	1753.84/ 2+	GSIIGYAYIGSMCHPK				

Protein no.	Peptide mass/ charge	Peptide sequence	Sequence coverage (%)	Protein name	Acc. no.	Theor. mass /theor. PI
	965.57/2+	KTYLLAEK				
6	653.37/2+	DIIHR	24.46	Acutusin 2	D2Y164	46662/5.51
	1089.58/2+	DLINVETSAK				
	1450.78/3+	ENPPCILNKPLR				
	1238.58/2+	NTLESFGEWR				
	1498.61/2+	YFSDCSYIQCR				
	639.33/2+	EYISK				
	3303.59/3+	ISHDNAQLLTATDLDGPTIGLAYVASM*CDPK				
	3287.59/3+	ISHDNAQLLTATDLDGPTIGLAYVASMCDPK				
	2001.02/2+	YLYIDTSVAAIEIWSEK				
	3071.58/3+	YLYIDTSVAAIEIWSEKDLINVETSAK				
7	767.37/2+	STEFQR	16.83	Snake venom metalloproteinase Ac1	Q7LZS9	22945/5.48
	653.37/2+	DIIHR				
	1181.6/2+	ENPPCILNKP				
	781.36/2+	SFGEWR				
	852.5/2+	AKDLIHR				
	639.33/2+	EYISK				
	1801.92/3+	EYISKENPPCILNKP				
8	781.36/2+	SFGEWR	10.29	Metalloprotease BOJUMET III	Q7T1T3	26887/5.53

Protein no.	Peptide mass/ charge	Peptide sequence	Sequence coverage (%)	Protein name	Acc. no.	Theor. mass /theor. PI
	1015.46/ 2+	ARGDNPDDR				
	1151.46/ 2+	CTGQSADCPR				
	788.32/2 +	GDNPDDR				
9	767.45/2 +	HSVGIVR	4.97	Snake venom metalloproteinase (Type II) 4	J3RY7 2	54231/5. 46
	1137.44/ 2+	CTGQSGDCPR				
	788.32/2 +	GDNPDDR				
	1906.74/ 3+	GDNPDDRCTGQSGDCPR				
10	765.36/2 +	TEICSR	4.81	Snake venom metalloproteinase (Type II) 5h	J3S3W 0	53647/5. 52
	1137.44/ 2+	CTGQSGDCPR				
	788.32/2 +	GDNPDDR				
	1906.74/ 3+	GDNPDDRCTGQSGDCPR				
11	735.35/2 +	EGTICR	3.94	Snake venom metalloproteinase (Type II) 6	J3S828	54204/5. 44
	767.45/2 +	HSVGIVR				
	765.36/2 +	TEICSR				
12	1845.92/ 2+	SHDNVQLLTGMIFNEK	3.51	Zinc metalloproteinase homolog-disintegrin albolatin	P0C6B 6	54264/5. 18
	1974.01/ 3+	KSHDNVQLLTGMIFNEK				
13	653.37/2 +	DIIHR	50	Zinc metalloproteinase /disintegrin	Q9IA X6	52517/5. 41

Protein no.	Peptide mass/ charge	Peptide sequence	Sequence coverage (%)	Protein name	Acc. no.	Theor. mass /theor. PI
	1089.58/ 2+	DLINVETSAK				
	1450.78/ 3+	ENPPCILNKPLR				
	1892.78/ 2+	LNQGAQCTAGPCCDQGR				
	1238.58/ 2+	NTLESFGEWR				
	1429.68/ 2+	TWIYEM*SNTIR				
	1413.68/ 2+	TWIYEMSNTIR				
	2194.89/ 2+	YFSDCSYIQCWDYIM*K				
	2178.9/2 +	YFSDCSYIQCWDYIMK				
	798.33/2 +	YNGDSDK				
	1139.55/ 2+	FKEEGTICR				
	1928.74/ 2+	GDDLDDYCNGISGDCPR				
	2889.16/ 3+	HDEGSCSCGSGYTCIM*SPVINPDAMK				
	2873.17/ 3+	HDEGSCSCGSGYTCIMSPVINPDAMK				
	3303.59/ 3+	ISHDNAQLLTATDLDGPTIGLAYVASM *CDPK				
	3287.59/ 3+	ISHDNAQLLTATDLDGPTIGLAYVASM CDPK				
	3172.7/5 +	SVGVVQDHSSVNHLVAITLAHEIAHNL GVR				
	2001.02/ 2+	YLYIDTSVAAIEIWSEK				
	3071.58/ 3+	YLYIDTSVAAIEIWSEKDLINVETSAK				
	1859.89/ 3+	YM*EIVIVVDHSM*YTK				

Protein no.	Peptide mass/ charge	Peptide sequence	Sequence coverage (%)	Protein name	Acc. no.	Theor. mass /theor. PI
	1843.9/2+	YM*EIVIVVDHSMYTK				
	1827.9/3+	YMEIVIVVDHSMYTK				
14	1875.89/2+	AWVYEMINTITESYR	40.08	Zinc metalloproteinase /disintegrin	Q9PWJ0	53890/5.3
	653.37/2+	DIIHR				
	1546.8/2+	DLINVETSAENTLK				
	1450.78/3+	ENPPCILNKPLR				
	1892.78/2+	LNQGAQCTAGPCCDQGR				
	781.36/2+	SFGEWR				
	2194.89/2+	YFSDCSYIQCWDYIM*K				
	2178.9/2+	YFSDCSYIQCWDYIMK				
	798.33/2+	YNGDSDK				
	852.5/2+	AKDLIHR				
	2169.89/3+	ARGDDLDDYCNGISADCPR				
	1139.55/2+	FKEEGTICR				
	2889.16/3+	HDEGSCSCGSGYTCIM*SPVINPDAMK				
	2873.17/3+	HDEGSCSCGSGYTCIMSPVINPDAMK				
	3303.59/3+	ISHDNAQLLTATDLDGPTIGLAYVASM*CDPK				
	3287.59/3+	ISHDNAQLLTATDLDGPTIGLAYVASMCDPK				
	1397.71/3+	SVGIVQDHSSVNR				

Protein no.	Peptide mass/ charge	Peptide sequence	Sequence coverage (%)	Protein name	Acc. no.	Theor. mass /theor. PI
15	653.37/2+	DIIHR	10.41	Zinc metalloproteinase /disintegrin	Q90WC0	35109/5.4
	735.35/2+	EGTICR				
	798.33/2+	YNGDSDK				
	1812.69/2+	QGAQCAEGLCCDQCR				
16	735.35/2+	EGTICR	7.68	Zinc metalloproteinase /disintegrin	Q9PVK9	53409/4.76
	3336.62/4+	ISHDNAQLLTTIDLDGNTIGLAHVGTM*CDPK				
17	653.37/2+	DIIHR	2.9	Zinc metalloproteinase /disintegrin	Q7SZE0	50411/5.01
	1015.46/2+	ARGDNPDDR				
	788.32/2+	GDNPDDR				
18	1015.46/2+	ARGDNPDDR	3.35	Metalloprotease PIIa	V5IWE4	53648/5.15
	788.32/2+	GDNPDDR				
	856.48/2+	LHFVANR				
19	1311.74/2+	FIELVIVADHR	5.37	Zinc metalloproteinase -disintegrin stejnitin	P0DM87	54436/5.22
	1746.85/3+	HDNAQLLTGM*IFNEK				
	1730.85/2+	HDNAQLLTGMIFNEK				
20	824.38/2+	YNGDLDK	6.53	Zinc metalloproteinase -disintegrin bilitoxin-1	P0C6E3	32313/5.28
	1065.56/2+	YNGDLDKIK				

Protein no.	Peptide mass/ charge	Peptide sequence	Sequence coverage (%)	Protein name	Acc. no.	Theor. mass /theor. PI
	1151.46/ 2+	CTGQSADCPR				
21	1230.52/ 2+	DNSPGQNNPCK	13.31	Zinc metalloproteinase -disintegrin-like jararhagin	P3043 1	63982/5. 21
	4005.62/ 3+	FSKSGTECRASM*SECDPAEHCTGQSS ECPADVFHK				
	818.44/2 +	GM*VLPGTK				
	802.45/2 +	GMVLPGTK				
	1052.43/ 2+	GNYYGYCR				
	1301.51/ 3+	M*FYSNDDEHK				
	2100.94/ 3+	M*FYSNDDEHKGM*VLPGTK				
	1285.52/ 2+	MFYSNDDEHK				
	2068.95/ 3+	MFYSNDDEHKGMVLPGTK				
	2953.15/ 3+	ASM*SECDPAEHCTGQSSECPADVFHK				
	2937.15/ 4+	ASMSECDPAEHCTGQSSECPADVFHK				
	1794.79/ 3+	LYCKDNSPGQNNPCK				
22	1230.52/ 2+	DNSPGQNNPCK	12.48	Zinc metalloproteinase -disintegrin-like VAP2B	Q9028 2	68247/5. 03
	4005.62/ 3+	FSKSGTECRASM*SECDPAEHCTGQSS ECPADVFHK				
	818.44/2 +	GM*VLPGTK				
	802.45/2 +	GMVLPGTK				
	1052.43/ 2+	GNYYGYCR				

Protein no.	Peptide mass/ charge	Peptide sequence	Sequence coverage (%)	Protein name	Acc. no.	Theor. mass /theor. PI
	2114.95/ 3+	M*FYSNEDEHKGM*VLPGTK				
	2953.15/ 3+	ASM*SECDPAEHCTGQSSECPADVFHK				
	2937.15/ 4+	ASMSECDPAEHCTGQSSECPADVFHK				
	1794.79/ 3+	LYCKDNSPGQNNPCK				
23	2401.96/ 3+	LRPGTQCEDGECCEQCQFK	4.58	SVMP-CohPH-1	T1DM N4	67871/5. 27
	1012.51/ 2+	YTSNSTAIR				
24	1231.5/2 +	DDSPGQNNPCK	27.7	Zinc metalloproteinase -disintegrin-like acurhagin	Q9W6 M5	68542/5. 03
	818.44/2 +	GM*VLPGTK				
	802.45/2 +	GMVLPGTK				
	1052.43/ 2+	GNYYGYCR				
	1499.76/ 3+	HDNAQLITGIDFR				
	1018.49/ 2+	IPCASEDVK				
	2138.09/ 3+	ITVKPDVDYTLNAFAEWR				
	1627.86/ 3+	KHDNAQLITGIDFR				
	1146.58/ 2+	KIPCASEDVK				
	1301.51/ 3+	M*FYSNDDEHK				
	2100.94/ 3+	M*FYSNDDEHKGM*VLPGTK				
	1684.64/ 2+	M*YEAANNM*NEM*YR				
	1668.65/ 2+	M*YEAANNM*NEMYR				

Protein no.	Peptide mass/ charge	Peptide sequence	Sequence coverage (%)	Protein name	Acc. no.	Theor. mass /theor. PI
	1652.65/ 2+	M*YEAANNMNEMYR				
	1285.52/ 2+	MFYSNDDEHK				
	2068.95/ 3+	MFYSNDDEHKGMVLPGTK				
	1668.65/ 2+	MYEAANNM*NEM*YR				
	1652.65/ 2+	MYEAANNM*NEMYR				
	1636.66/ 2+	MYEAANNMNEMYR				
	837.47/2 +	TYLLAEK				
	1582.66/ 2+	VCSNGHCVDVTTAY				
	824.38/2 +	YNGDLDK				
	1065.56/ 2+	YNGDLDKIK				
	2953.15/ 3+	ASM*SECDPAEHCTGQSSECPADVFHK				
	2937.15/ 4+	ASMSECDPAEHCTGQSSECPADVFHK				
	1753.84/ 2+	GSIIGYAYIGSMCHPK				
	965.57/2 +	KTYLLAEK				
	1795.77/ 2+	LYCKDDSPGQNNPCK				
25	1230.52/ 2+	DNSPGQNNPCK	24.55	Metalloproteinas e	V5Z14 1	67765/5. 22
	2064.86/ 2+	DYCSCGANSCVMSATLIR				
	1044.44/ 2+	FSTSGTECR				
	818.44/2 +	GM*VLPGTK				

Protein no.	Peptide mass/ charge	Peptide sequence	Sequence coverage (%)	Protein name	Acc. no.	Theor. mass /theor. PI
	802.45/2+	GMVLPGTK				
	1274.5/2+	M*FYSSDDEHK				
	1258.5/2+	MFYSSDDEHK				
	2057.93/3+	MFYSSDDEHKGM*VLPGTK				
	2041.94/3+	MFYSSDDEHKGMVLPGTK				
	2824.14/4+	SECDLPEYCTGQSIHCPTDDFHR				
	1783.63/3+	SGSQCGHGECCEQCK				
	2809.05/3+	SGSQCGHGECCEQCKFSTSGTECR				
	1582.66/2+	VCSNGHCVDVTTAY				
	1013.51/2+	VPCAPQDVK				
	1794.79/3+	LYCKDNSPGQNNPCK				
	1871.82/2+	NGQPCLNNLGYCYNGK				
	1384.79/2+	VALTGLEIWLNR				
26	3239.22/4+	GAGTQCRAAM*DECDMADLCTGQSADCTDR	30.59	Zinc metalloproteinase-disintegrin-like agkihagin	Q1PS45	67572/5.77
	818.44/2+	GM*VLPGTK				
	802.45/2+	GMVLPGTK				
	1058.53/2+	IACEPQNVK				
	1299.72/2+	IIVQPSADDTLK				
	1455.79/2+	LFLVADNIM*YLK				

Protein no.	Peptide mass/ charge	Peptide sequence	Sequence coverage (%)	Protein name	Acc. no.	Theor. mass /theor. PI
	1439.8/2+	LFLVADNIMYLK				
	1828.78/2+	QNPCNIYYSPSNEDK				
	2400.99/2+	SCVMAGTLSCEASYLFSDCSR				
	767.38/2+	SFATWR				
	4374.09/4+	SHDNAQLLTGINFNGPTAGLAYLGGICDPMYSTAIVQDHNK				
	2508.9/2+	AAM*DECDMADLCTGQSADCTDR				
	2492.91/2+	AAMDECDMADLCTGQSADCTDR				
	2114.06/3+	MNIHVAVTDLEIWSDKDK				
	1812.69/2+	QGAQCAEGLCCDQCR				
27	818.44/2+	GM*VLPGTK	7.68	Zinc metalloproteinase-disintegrin-like HV1	Q90ZI3	68191/5.97
	802.45/2+	GMVLPGTK				
	1058.53/2+	IACEPQNVK				
	1858.73/2+	NGQPCQNNNGYCYNR				
	1812.69/2+	QGAQCAEGLCCDQCR				
28	760.44/2+	DIMLIR	8.08	Venom serine proteinase-like protein 1	Q6T6S7	28982/8.22
	1593.76/2+	TLCAGILEGGIDSCK				
29	760.44/2+	DIMLIR	13.46	Snake venom serine protease homolog	Q7T229	28654/8.81
	888.43/2+	FFCLSSK				

Protein no.	Peptide mass/ charge	Peptide sequence	Sequence coverage (%)	Protein name	Acc. no.	Theor. mass /theor. PI
	1118.59/ 2+	TLCAGILEGGK				
30	760.44/2 +	DIMLIR	11.63	Kallikrein-CohID-4	T1DM M6	28204/5. 64
	1118.59/ 2+	TLCAGILEGGK				
	1019.56/ 3+	DKDIM*LIR				
	1003.56/ 2+	DKDIMLIR				
	1190.62/ 2+	IMGWGTISPTK				
31	760.44/2 +	DIMLIR	13.9	Kallikrein-CohID-1	T1E6T 7	28248/5. 05
	1961.03/ 3+	HIAPISLPSSPPSVGSVCR				
	1118.59/ 2+	TLCAGILEGGK				
32	760.44/2 +	DIMLIR	29.62	Thrombin-like enzyme acutobin	Q9I8X 2	28815/5. 94
	1462.68/ 2+	FDDEQGREPIEK				
	1243.57/ 2+	VYDYNDWIR				
	1019.56/ 3+	DKDIM*LIR				
	1003.56/ 2+	DKDIMLIR				
	2448.18/ 3+	DVLPDVPHCVNINLVNNAECR				
	1754.76/ 2+	TLCAGVM*QGGIDSCNR				
	1738.77/ 2+	TLCAGVMQGGIDSCNR				
	1176.58/ 2+	VM*GWGAISPSR				
	1160.59/ 2+	VMGWGAISPSR				

Protein no.	Peptide mass/ charge	Peptide sequence	Sequence coverage (%)	Protein name	Acc. no.	Theor. mass /theor. PI
33	3738.76/ 3+	ETFPDVPYCANINLLDHAVCQAGYPEL LAEYR	45.96	Venom thrombin-like enzyme	A1E23 7	25519/5. 46
	1961.03/ 3+	HIAPISLPSSPPSVGSVCR				
	1001.56/ 3+	LDKPISNSK				
	1674.87/ 2+	NNNEVLDKDIM*LIK				
	1658.88/ 3+	NNNEVLDKDIMLIK				
	1204.64/ 2+	IM*GWGSITPVK				
	1188.64/ 2+	IMGWGSITPVK				
	1230.54/ 2+	NIAGNTDATCPP				
	1103.59/ 2+	TLCAGIVQGGK				
34	3782.76/ 4+	DILPDVPHCANINLLNYM*ECVAHYPD VPETTR	43.08	Snake venom serine protease Da-36	J7LCB 0	29057/6. 05
	3766.77/ 4+	DILPDVPHCANINLLNYMECVAHYPDV PETTR				
	686.44/2 +	DVVLLK				
	1129.66/ 2+	WEKDVVLLK				
	3842.82/ 3+	LLCAGVLEGGIDTCNQDSGGPLICDGQ FQGIVFFGK				
	1212.58/ 2+	VMGWGIM*SSTK				
	1196.58/ 2+	VMGWGIMSSTK				
	1734.88/ 2+	VSNYNDWIQNIIAGK				
	1734.88/ 2+	VSNYNDWIQNIIAGK				
	1243.57/ 3+	YAIEEHYYR				

Protein no.	Peptide mass/ charge	Peptide sequence	Sequence coverage (%)	Protein name	Acc. no.	Theor. mass /theor. PI
35	1491.76/ 2+	DILPDVPHCANIK	24.23	Thrombin-like enzyme 2	Q5I2C 5	29046/7. 07
	1115.61/ 2+	WENDVVLLK				
	2163.12/ 3+	STHIAPLSLPSSPPSIGSVCR				
	1212.58/ 2+	VMGWGIM*SSTK				
	1196.58/ 2+	VMGWGIMSSTK				
	1243.57/ 3+	YAIEEHYYR				
36	1491.76/ 2+	DILPDVPHCANIK	16.15	Thrombin-like protein 1	Q5I2B 6	29158/7. 52
	686.44/2 +	DVVLLK				
	1129.66/ 2+	WEKDVVLLK				
	1212.58/ 2+	VMGWGIM*SSTK				
	1196.58/ 2+	VMGWGIMSSTK				
	1243.57/ 3+	YAIEEHYYR				
37	686.44/2 +	DVVLLK	15.77	Thrombin-like protein 3	Q5I2B 5	29071/6. 12
	1129.66/ 2+	WEKDVVLLK				
	1230.54/ 2+	NIAGNTDATCPP				
	1212.58/ 2+	VMGWGIM*SSTK				
	1196.58/ 2+	VMGWGIMSSTK				
	1243.57/ 3+	YAIEEHYYR				
38	888.43/2 +	FFCLSSK	35.74	Venom thrombin-like enzyme	A1E2S 1	25179/5. 87

Protein no.	Peptide mass/ charge	Peptide sequence	Sequence coverage (%)	Protein name	Acc. no.	Theor. mass /theor. PI
	4125.81/ 4+	GSCDGDSGGPLICNGEIQGIVSWGGDIC AQPHEPGHYTK				
	1118.59/ 2+	TLCAGILEGGK				
	1089.58/ 2+	WGNDIMLIK				
	1850.98/ 2+	IAPISLPSNPPSVGSVCR				
39	4125.81/ 4+	GSCDGDSGGPLICNGEIQGIVSWGGDIC AQPHEPGHYTK	27.23	Venom thrombin-like enzyme	Q90Z4 7	25255/5. 16
	1674.87/ 2+	NNNEVLDKDIM*LIK				
	1658.88/ 3+	NNNEVLDKDIMLIK				
	1118.59/ 2+	TLCAGILEGGK				
40	1511.7/2 +	VIGGNECNINEHR	8.05	Thrombin-like protein	A8HR 02	26006/5. 95
	760.44/2 +	DIMLIR				
41	1512.69/ 2+	VIGGDECNINEHR	15.55	Alpha-fibrinogenase shedaoenase	Q6T5L 0	26416/6. 7
	760.44/2 +	DIMLIR				
	888.43/2 +	FFCLSSK				
	1118.59/ 2+	TLCAGILEGGK				
42	760.44/2 +	DIMLIR	15.95	Snake venom serine protease serpentokallikrei n-2	Q9DG 84	28320/6. 58
	1118.59/ 2+	TLCAGILEGGK				
	1639.76/ 2+	CANINLLDYEVCR				
	1190.62/ 2+	IMGWGTISPTK				

Protein no.	Peptide mass/ charge	Peptide sequence	Sequence coverage (%)	Protein name	Acc. no.	Theor. mass /theor. PI
43	666.36/2+	CFVLK	57.42	Snaclec agkicetin-C subunit alpha	Q9DEA2	17798/8.83
	837.37/2+	HVFM*CK				
	821.38/2+	HVFMCK				
	906.4/2+	TWEDAER				
	1411.71/2+	VSYENLVEPFSK				
	1539.81/2+	VSYENLVEPFSKK				
	676.41/2+	YLKPR				
	1491.77/2+	EADFVAGVLSENIK				
	2009.91/3+	FCTEQANGGHLVSFESAR				
	1269.59/2+	TWENVYCGLK				
44	948.44/2+	FCTQQHK	67.81	Snaclec agkisacutacin subunit B	Q8JIW1	16726/5.26
	2660.32/3+	GSHLASFHSSEEADFVVTLTTPSLK				
	995.49/2+	LDYKDWR				
	1240.56/2+	NIWNGCYWK				
	1044.61/2+	TDLVWIGLK				
	820.38/2+	TWADAEK				
	693.32/2+	WSDGTK				
	1167.55/2+	EQFECLVSR				
	2424.99/2+	TVNNEWLSM*DCGTTCSFVCK				

Protein no.	Peptide mass/ charge	Peptide sequence	Sequence coverage (%)	Protein name	Acc. no.	Theor. mass /theor. PI
	2409/2+	TVNNEWLSMDCGTTCSFVCK				
45	795.41/2+	CFVLEK	61.69	Snaclec agglucetin subunit alpha-1	Q8JIV9	17317/5.44
	937.46/2+	FCTERPK				
	1311.67/2+	GGHLVSIESAGER				
	2904.19/2+	GQQCSTEWTDGSSVSYENFSEYQSK				
	1173.63/2+	QTDNVWLGLK				
	1782.81/3+	TWDDAEKFCTERPK				
	1249.64/2+	DFVAQLVSENK				
	1961.89/2+	TWLNLNCGSEYAFVCK				
46	2219.04/3+	ECHLVSFHSAEEVDFVVSK	51.61	Akitonin	Q90WL9	14658/4.87
	961.51/2+	KTWAEAQK				
	995.49/2+	LDYKDWR				
	1240.56/2+	NIWNGCYWK				
	833.42/2+	TWAEAQK				
	693.32/2+	WSDGTK				
	1167.55/2+	EQFECLVSR				
	840.37/2+	FCTEQR				
47	713.38/2+	CLGVHK	50.66	ACF 1/2 A-chain	Q8JIW0	17108/6.51
	2763.17/2+	QCSIEWSDGSSISYENWIEEESK				

Protein no.	Peptide mass/ charge	Peptide sequence	Sequence coverage (%)	Protein name	Acc. no.	Theor. mass /theor. PI
	2891.27/ 3+	QCSIEWSDGSSISYENWIEEESKK				
	2706.38/ 4+	QVNGGHLVSIESSGEADFVAHLIAQK				
	1315.56/ 2+	TWADAESFCTK				
	1331.55/ 2+	WENFYCEQR				
	1459.64/ 2+	KWENFYCEQR				
48	1048.56/ 3+	GGHLVSVHSR	41.61	Snaclec agglucetin subunit beta-2	Q8AYA3	17234/8.1
	1961.03/ 3+	HIAPISLPSSPPSVGSVCR				
	1001.56/ 3+	LDKPISNSK				
	1674.87/ 2+	NNNEVLDKDIM*LIK				
	1658.88/ 3+	NNNEVLDKDIMLIK				
	1699.67/ 2+	TDGDNQWLNM*DCSK				
	1683.67/ 2+	TDGDNQWLNMDCSK				
	1685.72/ 2+	TWDDAEKFCTEQR				
	1325.64/ 2+	EWSDGTKLDFK				
	840.37/2+	FCTEQR				
	1204.64/ 2+	IM*GWGSITPVK				
	1188.64/ 2+	IMGWGSITPVK				
	992.47/2+	KTWDDAEK				
	1230.54/ 2+	NIAGNTDATCPP				

Protein no.	Peptide mass/ charge	Peptide sequence	Sequence coverage (%)	Protein name	Acc. no.	Theor. mass /theor. PI
	1103.59/ 2+	TLCAGIVQGGK				
	1627.76/ 2+	WSSYEGHCYLVVK				
49	1360.69/ 2+	AWSGIPECIISK	57.53	Snaclec agkicetin-C subunit beta	Q9DEA1	16688/5.66
	2219.04/ 3+	ECHLVSFHSAEEVDFVVSK				
	961.51/2 +	KTWAEAQK				
	3600.72/ 3+	TFPILSYDLVWIGLNNIWNDCMLEWSDGTK				
	1106.52/ 2+	TSDNQWISR				
	833.42/2 +	TWAEAQK				
	840.37/2 +	FCTEQR				
50	2049.94/ 2+	IWNQCNWQWSNAAMLK	65.07	Snaclec anticoagulant protein subunit B	Q9DEF8	16997/5.25
	2024/2+	LAFQTFDYGIFWMGLSK				
	3682.65/ 4+	NWADAENFCTQQHTGSHLVSFQSTEEADFVVK				
	1860.78/ 2+	YTDWAEESYCVYFK				
	1345.59/ 2+	M*IANFVCEFQA				
	1329.6/2 +	MIANFVCEFQA				
	636.31/2 +	SITCR				
51	1397.7/3 +	CLGVHIETGFHK	58.55	Snaclec agkisacutacin subunit A	Q9IAM1	17109/5.51
	2763.17/ 2+	QCSIEWSDGSSISYENWIEEESK				
	2891.27/ 3+	QCSIEWSDGSSISYENWIEEESKK				

Protein no.	Peptide mass/ charge	Peptide sequence	Sequence coverage (%)	Protein name	Acc. no.	Theor. mass /theor. PI
	2683.37/ 3+	QVNGGHLVSIESSGEADFVGQLIAQK				
	1345.57/ 2+	TWTDAESFCTK				
	2121.83/ 2+	WENFYCEQQDPFVCEA				
52	2603.06/ 3+	DCPSDWSSYEGHCYKPFNEPK	68.29	Anticogulant protein subunit beta	I2GAE 4	14554/5. 02
	2049.94/ 2+	IWNQCNWQWSNAAMLK				
	2024/2+	LAFQTFDYGIFWMGLSK				
	1860.78/ 2+	YTDWAEESYCVYFK				
	1345.59/ 2+	M*IANFVCEFQA				
	1329.6/2 +	MIANFVCEFQA				
	636.31/2 +	SITCR				
53	705.33/2 +	CGALER	37.97	Snaclec agglucetin subunit alpha-2	Q8AY A5	17977/4. 92
	905.37/2 +	NWDDAER				
	2770.2/2 +	QCSTEWSDGSSVSYENLLELYM*R				
	2754.2/2 +	QCSTEWSDGSSVSYENLLELYMR				
	718.35/2 +	ETGFHK				
	1877.87/ 3+	FCTEQADGGHLVSIESK				
	833.43/2 +	KCGALER				
54	1981.77/ 3+	NWDDAESFCSGQHEGSR	40	Snaclec clone 2100755	Q8JIV 8	17944/6. 3
	826.41/2 +	SVSFVCK				

Protein no.	Peptide mass/ charge	Peptide sequence	Sequence coverage (%)	Protein name	Acc. no.	Theor. mass /theor. PI
	1094.45/ 2+	WEWSDDTR				
	1625.78/ 2+	YTSM*WLGLNNPWK				
	1609.78/ 2+	YTSMWLGLNNPWK				
	783.45/2 +	LASIHSR				
	1268.65/ 2+	RPYCTVM*VVK				
	1252.65/ 2+	RPYCTVMVVK				
55	666.36/2 +	CFVLK	7.01	Snaclec mamushigin subunit alpha	Q9YG G9	18333/8. 66
	840.37/2 +	FCTEQR				
56	666.36/2 +	CFVLK	8.96	C-type lectin factor IX/X binding protein A subunit	T2HP A6	15512/8. 45
	883.4/2+	FCTEQAK				
57	948.44/2 +	FCTQQHK	10.14	Snaclec stejaggregin-A subunit beta-1	Q71R Q0	17019/5. 51
	992.47/2 +	KTWDDAEK				
58	1343.55/ 2+	CTGQDCYGGVAR	16.16	Snake venom 5'-nucleotidase	B6EW W8	64433/8. 65
	1718.87/ 2+	ETPVLSNPGPYLEFR				
	2689.34/ 3+	ETPVLSNPGPYLEFRDEVEELQK				
	1110.54/ 2+	QAFEHSVHR				
	874.48/2 +	VFPAVEGR				
	951.55/2 +	VGIIGYTTK				

Protein no.	Peptide mass/ charge	Peptide sequence	Sequence coverage (%)	Protein name	Acc. no.	Theor. mass /theor. PI
	851.46/2+	VVYDLSR				
	1569.81/2+	HGQGM*GELLQVSGIK				
	1553.81/3+	HGQGMGELLQVSGIK				
	1449.72/2+	VVSLNVLCTECR				
59	1026.59/2+	ASGNPILLNK	12.5	5'-nucleotidase	W8EFS0	45030/6.45
	1718.87/2+	ETPVLSNPGPYLEFR				
	1110.54/2+	QAFEHSVHR				
	874.48/2+	VFPAVEGR				
	951.55/2+	VGIIGYTTK				
60	1026.59/2+	ASGNPILLNK	10.98	Ecto-5'-nucleotidase 1	U3FYP9	62982/8.93
	1718.87/2+	ETPVLSNPGPYLEFR				
	2689.34/3+	ETPVLSNPGPYLEFRDEVEELQK				
	1110.54/2+	QAFEHSVHR				
	951.55/2+	VGIIGYTTK				
	1449.72/2+	VVSLNVLCTECR				
61	1026.59/2+	ASGNPILLNK	13.89	5'-nucleotidase	U3T7C6	55516/6.98
	1110.54/2+	QAFEHSVHR				
	874.48/2+	VFPAVEGR				
	951.55/2+	VGIIGYTTK				

Protein no.	Peptide mass/ charge	Peptide sequence	Sequence coverage (%)	Protein name	Acc. no.	Theor. mass /theor. PI
	851.46/2+	VVYDLSR				
	1569.81/2+	HGQGM*GELLQVSGIK				
	1553.81/3+	HGQGMGELLQVSGIK				
	1449.72/2+	VVSLNVLCTECR				
62	1026.59/2+	ASGNPILLNK	17.6	5' -nucleotidase	T2HRS9	57090/8.27
	1111.53/2+	EAFEHSVHR				
	1718.87/2+	ETPVLSNPGPYLEFR				
	1555.82/3+	IIALGHSGFLEDQR				
	951.55/2+	VGIIGYTTK				
	851.46/2+	VVYDLSR				
	1569.81/2+	HGQGM*GELLQVSGIK				
	1553.81/3+	HGQGMGELLQVSGIK				
	1449.72/2+	VVSLNVLCTECR				
63	1355.66/2+	AATYFWPGSEVK	14.57	Venom phosphodiesterase 2	J3SBP3	91751/8.39
	2332.06/2+	EQSSPLSCPFGPVPSPDVSGCK				
	1260.7/2+	TFLPIFVNPVN				
	1589.79/2+	VNLMVDQQWMAVR				
	2816.54/2+	DVELLTGLNFYSGLKQPLPETLQLK				
	2723.27/3+	NLHNCVNLILLADHGMEEISCDR				

Protein no.	Peptide mass/ charge	Peptide sequence	Sequence coverage (%)	Protein name	Acc. no.	Theor. mass /theor. PI
64	1355.66/ 2+	AATYFWPGSEVK	14.62	Phosphodiesteras e	U3TBJ 5	100878/ 6.61
	2182.05/ 3+	RPDFYTLYIEEPDTTGHK				
	1260.7/2 +	TFLPIFVNPVN				
	1589.79/ 2+	VNLMVDQQWMAVR				
	1837.88/ 2+	AGYLESWDSLMPNINK				
	2816.54/ 2+	DVELLTGLNFYSGLKQPLPETLQLK				
	2723.27/ 3+	NLHNCVNLILLADHGMEEISCDR				
65	1338.73/ 2+	SLIQFETLIM*K	64.23	Acidic phospholipase A2	Q7SID 6	14032/4. 68
	1322.74/ 2+	SLIQFETLIMK				
	981.38/2 +	EICECDR				
	2684.05/ 3+	M*DSYTYSEENGDIVCGGDDPCKR				
	2511.96/ 2+	MDSYTYSEENGDIVCGGDDPCK				
	2668.06/ 3+	MDSYTYSEENGDIVCGGDDPCKR				
	776.36/2 +	VTGCDPK				
	1505.54/ 2+	CCFVHDCCYGK				
	1662.7/2 +	DNLDTYNSDTYWR				
	822.43/2 +	VAAVCFR				
66	850.46/2 +	AFAICLR	39.86	Basic phospholipase A2 homolog acutohaemolysin	O5738 5	15777/8. 82

Protein no.	Peptide mass/ charge	Peptide sequence	Sequence coverage (%)	Protein name	Acc. no.	Theor. mass /theor. PI
	1008.48/ 2+	M*IWQETGK				
	1446.74/ 3+	M*IWQETGKNPVK				
	992.49/2 +	MIWQETGK				
	1430.75/ 3+	MIWQETGKNPVK				
	1761.6/2 +	NQPCM*QEM*CECDK				
	1745.61/ 2+	NQPCM*QEMCECDK				
	1729.61/ 2+	NQPCMQEMCECDK				
	1546.65/ 2+	NYGLYGCNCGVGGR				
	973.46/2 +	GEPLDATDR				
67	2065.75/ 2+	DATDSCCFVHDCCYQK	48.55	Basic phospholipase A2 DAV-N6	Q1ZY 03	15853/8. 62
	981.38/2 +	EICECDR				
	1520.71/ 2+	TGVIICGEGTPCEK				
	1257.59/ 2+	WDIYPYSWK				
	1399.61/ 2+	YM*FYPDFLCK				
	1383.61/ 2+	YMFYPDFLCK				
	1173.6/2 +	AAAVCLGENLR				
68	981.38/2 +	EICECDR	27.87	Acidic phospholipase A2	P8465 1	13901/4. 95
	776.36/2 +	VTGCDPK				
	1505.54/ 2+	CCFVHDCCYGK				

Protein no.	Peptide mass/ charge	Peptide sequence	Sequence coverage (%)	Protein name	Acc. no.	Theor. mass /theor. PI
	1015.49/ 2+	GRPQDATDR				
69	735.35/2 +	EGTICR	52.08	Disintegrin accutin	P0DM 77	5333/6.1 8
	2169.89/ 3+	ARGDDLDDYCNGISADCPR				
70	2516.87/ 2+	EAGEECDCGSPENPCCDAATCK	56.16	Trimucrin	Q7T1S 0	7876/6.4 2
	1015.46/ 2+	ARGDNPDDR				
	1151.46/ 2+	CTGQSADCPR				
	788.32/2 +	GDNPDDR				
71	1293.6/2 +	EGWYANLGPMR	9.33	L-amino-acid oxidase	Q90W 54	57091/6. 53
	882.48/2 +	IFLTCTK				
	1514.7/2 +	ETDYEEFLEIAR				
	1958.94/ 2+	FDEIVGGMDKLPTSMYR				
72	1459.68/ 2+	YIAATDHEPTDAR	4.38	Aminopeptidase A	T2HQ 95	109903/ 5.66
	1064.57/ 2+	SQDVFTVLR				
	1190.6/2 +	VNYEPQVWR				
	1093.49/ 2+	YPDAGAGEASR				
73	1537.67/ 2+	MEWYPEAAANAER	100	Helicopsin	P0DJG 8	2620/4.7 8
	1100.58/ 2+	YTQIVWYK				
74	1737.85/ 2+	DLHYATVYWLEAEK	11.78	Phospholipase B-like 1	V8ND 68	58279/8. 1
	835.37/2 +	DDPFWR				

Protein no.	Peptide mass/ charge	Peptide sequence	Sequence coverage (%)	Protein name	Acc. no.	Theor. mass /theor. PI
	1331.54/ 2+	HNPCNTICCR				
	1191.55/ 2+	NNKDDPFWR				
	1889.87/ 2+	QNSGTYNNQYMILDTK				
	1037.48/ 2+	IANMMADSGK				
75	835.37/2 +	DDPFWR	24.05	Phospholipase b	T2HP6 8	64233/8. 74
	1331.54/ 2+	HNPCNTICCR				
	1191.55/ 2+	NNKDDPFWR				
	1889.87/ 2+	QNSGTYNNQYMILDTK				
	1011.48/ 2+	TWAETFEK				
	905.48/2 +	VADISMAAK				
	1332.69/ 2+	VVPESLFAWER				
	1037.48/ 2+	IANMMADSGK				
	2312.14/ 2+	NAGYVIAQLDGLYMGNVEWAK				
	1600.74/ 2+	NGYWPSYNIPFDK				
	1704.91/ 2+	SLEDGTLYIIEQVPK				
	1404.64/ 2+	YNNYKEDPYAK				
76	827.43/2 +	FASFIDK	6.77	Uncharacterized protein	G1KH 77	61267/7. 51
	1277.71/ 2+	LALDIEIATYR				
	1152.55/ 2+	NKYEDEINK				

Protein no.	Peptide mass/ charge	Peptide sequence	Sequence coverage (%)	Protein name	Acc. no.	Theor. mass /theor. PI
	1308.65/ 3+	NKYEDEINKR				
	1208.62/ 2+	ATAENDFVTLK				
77	1090.53/ 2+	VTMQNLNDR	5.93	Uncharacterized protein	R4GAI 6	48910/4. 91
	807.4/2+	LAADDFR				
	1201.62/ 2+	QSVEADINGLR				
78	807.4/2+	LAADDFR	3.49	Uncharacterized protein	G1KU G5	50674/5. 07
	1029.59/ 2+	VLDELTLAR				
79	1139.54/ 2+	DYQELMNVK	2.79	Uncharacterized protein	R4GB 02	66639/7. 1
	1033.52/ 2+	TLLEGEESR				
80	880.45/2 +	AALFTDSR	2.21	Uncharacterized protein	H9GF 03	76096/5. 23
	965.48/2 +	YWIQAER				
81	795.47/2 +	IIAPPER	7.96	Actin, alpha skeletal muscle	T1E7F 6	42051/5. 23
	1500.71/ 3+	QEYDEAGPSIVHR				
	1130.55/ 2+	GYSFVTTAER				
82	1363.64/ 2+	ALTMEGNQASWR	9.47	Venom nerve growth factor	Q3I5F 4	27382/7. 59
	1293.63/ 2+	IDTACVCVISR				
83	1442.73/ 2+	LIFFDGEEAFVR	35.87	Glutaminyl-peptide cyclotransferases	M9NC I9	42295/8. 52
	1064.54/ 2+	YFPPQLDGK				
	1474.74/ 2+	HPVEDDHIPFLR				

Protein no.	Peptide mass/ charge	Peptide sequence	Sequence coverage (%)	Protein name	Acc. no.	Theor. mass /theor. PI
	1664.86/ 3+	MWQNDLHPILIER				
	1770.87/ 2+	NLYDLGLLNNYSSER				
	1537.82/ 2+	NPVFPVYFLNTAR				
	1518.85/ 2+	TFSNIISTLNPLAK				
	2628.22/ 3+	VWHTMEDNEENLDKPTIDNISK				
	1254.57/ 2+	WSPSDSLYGSR				
	1153.56/ 2+	YPGSPGSYAVR				
84	985.56/2 +	EPAISLVEK	2.19	Vascular non-inflammatory molecule 2	V8N7 Y3	97658/5. 33
	1157.64/ 2+	NLDILEEAIK				
85	1138.5/2 +	SVDFDSESPR	45.91	Cysteine-rich seceretory protein Da-CRPa	F2Q6 G0	24743/5. 96
	1540.66/ 2+	CGENIYMSPNPMK				
	1537.67/ 2+	MEWYPEAAANAER				
	1795.79/ 2+	SLLQQDSCQDAGMQSK				
	2436.2/3 +	YGVGADPPNAVTGHYTQIVWYK				
	1905.02/ 3+	KPEIQNEIVDLHNSLR				
	1161.59/ 2+	SVNPTASNMLK				
