# Supplementary material for: Exploring the five-paced viper (Deinagkistrodon acutus) venom proteome by integrating a combinatorial peptide ligand library approach with shotgun LC-MS/MS
Source: J Venom Anim Toxins Incl Trop Dis. 2021 Oct 25;27:e20200196. doi: 10.1590/1678-9199-JVATITD-2020-0196 (PMC8547348; doi:10.1590/1678-9199-JVATITD-2020-0196)
Supplement: Additional file 2. [file 1678-9199-jvatitd-27-e20200196-s2.pdf]

*J Venom Anim Toxins incl Trop Dis*
https://doi.org/10.1590/1678-9199-JVATITD-2020-0196

# Supplementary Material to “Exploring the five-paced viper (*Deinagkistrodon acutus*) venom proteome by integrating a combinatorial peptide ligand library approach with shotgun LC-MS/MS”

**Additional file 2**. Proteins identified from *D. acutus* venom.

| No. | Protein name | Acc. no. | Theoretical mass/PI | Similar venom compound | | Function classified |
|---|---|---|---|---|---|---|
| | | | | Species | Evidence | |
| 1 | Snake venom metalloproteinase AaPA | Q2EI26 | 46757/5.3 | *Deinagkistrodon acutus* | T | Metalloprotease P-I |
| 2 | Acutusin 1 | D2Y163 | 46675/5.46 | *Deinagkistrodon acutus* | T | Metalloprotease P-I |
| 3 | Snake venom metalloproteinase aculysin-1 | Q9W7S2 | 47304/5.89 | *Deinagkistrodon acutus* | T | Metalloprotease P-I |
| 4 | Snake venom metalloproteinase H5 | Q9IAY2 | 46033/5.75 | *Deinagkistrodon acutus* | P | Metalloprotease P-I |
| 5 | Recombinant fibrinogenase II | A2TK72 | 26712/5.14 | *Deinagkistrodon acutus* | T | Metalloprotease P-I |
| 6 | Acutusin 2 | D2Y164 | 46662/5.51 | *Deinagkistrodon acutus* | T | Metalloprotease P-I |
| 7 | Snake venom metalloproteinase Ac1 | Q7LZS9 | 22945/5.48 | *Deinagkistrodon acutus* | P | Metalloprotease P-I |
| 8 | Metalloprotease BOJUMET III | Q7T1T3 | 26887/5.53 | *Bothrops jararacussu* | T | Metalloprotease P-II |
| 9 | Snake venom metalloproteinase (Type II) 4 | J3RY72 | 54231/5.46 | *Crotalus adamanteus* | T | Metalloprotease P-II |
| 10 | Snake venom metalloproteinase (Type II) 5h | J3S3W0 | 53647/5.52 | *Crotalus adamanteus* | T | Metalloprotease P-II |

| No. | Protein name | Acc. no. | Theoretical mass/PI | Similar venom compound | | Function classified |
|---|---|---|---|---|---|---|
| | | | | Species | Evidence | |
| 11 | Snake venom metalloproteinase (Type II) 6 | J3S828 | 54204/5.44 | *Crotalus adamanteus* | T | Metalloprotease P-II |
| 12 | Zinc metalloproteinase homolog-disintegrin albolatin | P0C6B6 | 54264/5.18 | *Cryptelytrops albolabris* | P | Metalloprotease P-II |
| 13 | Zinc metalloproteinase/disintegrin | Q9IAX6 | 52517/5.41 | *Deinagkistrodon acutus* | T | Metalloprotease P-II |
| 14 | Zinc metalloproteinase/disintegrin | Q9PWJ0 | 53890/5.3 | *Deinagkistrodon acutus* | P | Metalloprotease P-II |
| 15 | Zinc metalloproteinase/disintegrin | Q90WC0 | 35109/5.4 | *Gloydius brevicaudus* | P | Metalloprotease P-II |
| 16 | Zinc metalloproteinase/disintegrin | Q9PVK9 | 53409/4.76 | *Gloydius brevicaudus* | T | Metalloprotease P-II |
| 17 | Zinc metalloproteinase/disintegrin | Q7SZE0 | 50411/5.01 | *Gloydius saxatilis* | T | Metalloprotease P-II |
| 18 | Metalloprotease PIIa | V5IWE4 | 53648/5.15 | *Trimeresurus gracilis* | T | Metalloprotease P-II |
| 19 | Zinc metalloproteinase-disintegrin stejnitin | P0DM87 | 54436/5.22 | *Trimeresurus stejnegeri* | P | Metalloprotease P-II |
| 20 | Zinc metalloproteinase-disintegrin bilitoxin-1 | P0C6E3 | 32313/5.28 | *Agkistrodon bilineatus* | P | Metalloprotease P-II |
| 21 | Zinc metalloproteinase-disintegrin-like jararhagin | P30431 | 63982/5.21 | *Bothrops jararaca* | P | Metalloprotease P-III |
| 22 | Zinc metalloproteinase-disintegrin-like VAP2B | Q90282 | 68247/5.03 | *Crotalus atrox* | P | Metalloprotease P-III |
| 23 | SVMP-CohPH-1 | T1DMN4 | 67871/5.27 | *Crotalus oreganus helleri* | T | Metalloprotease P-III |
| 24 | Zinc metalloproteinase-disintegrin-like acurhagin | Q9W6M5 | 68542/5.03 | *Deinagkistrodon acutus* | P | Metalloprotease P-III |
| 25 | Metalloproteinase | V5Z141 | 67765/5.22 | *Deinagkistrodon acutus* | T | Metalloprotease P-III |

| No. | Protein name | Acc. no. | Theoretical mass/PI | Similar venom compound | | Function classified |
|---|---|---|---|---|---|---|
| | | | | Species | Evidence | |
| 26 | Zinc metalloproteinase-disintegrin-like agkihagin | Q1PS45 | 67572/5.77 | *Deinagkistrodon acutus* | T | Metalloprotease P-III |
| 27 | Zinc metalloproteinase-disintegrin-like HV1 | Q90ZI3 | 68191/5.97 | *Protobothrops flavoviridis* | P | Metalloprotease P-III |
| 28 | Venom serine proteinase-like protein 1 | Q6T6S7 | 28982/8.22 | *Bitis gabonica* | P | Serine protease |
| 29 | Snake venom serine protease homolog | Q7T229 | 28654/8.81 | *Bothrops jararacussu* | P | Serine protease |
| 30 | Kallikrein-CohID-4 | T1DMM 6 | 28204/5.64 | *Crotalus oreganus helleri* | T | Serine protease |
| 31 | Kallikrein-CohID-1 | T1E6T7 | 28248/5.05 | *Crotalus oreganus helleri* | T | Serine protease |
| 32 | Thrombin-like enzyme acutobin | Q9I8X2 | 28815/5.94 | *Deinagkistrodon acutus* | P | Serine protease |
| 33 | Venom thrombin-like enzyme | A1E237 | 25519/5.46 | *Deinagkistrodon acutus* | T | Serine protease |
| 34 | Snake venom serine protease Da-36 | J7LCB0 | 29057/6.05 | *Deinagkistrodon acutus* | H | Serine protease |
| 35 | Thrombin-like enzyme 2 | Q5I2C5 | 29046/7.07 | *Deinagkistrodon acutus* | T | Serine protease |
| 36 | Thrombin-like protein 1 | Q5I2B6 | 29158/7.52 | *Deinagkistrodon acutus* | T | Serine protease |
| 37 | Thrombin-like protein 3 | Q5I2B5 | 29071/6.12 | *Deinagkistrodon acutus* | T | Serine protease |
| 38 | Venom thrombin-like enzyme | A1E2S1 | 25179/5.87 | *Deinagkistrodon acutus* | T | Serine protease |
| 39 | Venom thrombin-like enzyme | Q90Z47 | 25255/5.16 | *Deinagkistrodon acutus* | T | Serine protease |
| 40 | Thrombin-like protein | A8HR02 | 26006/5.95 | *Deinagkistrodon acutus* | T | Serine protease |

| No. | Protein name | Acc. no. | Theoretical mass/PI | Similar venom compound | | Function classified |
|---|---|---|---|---|---|---|
| | | | | Species | Evidence | |
| 41 | Alpha-fibrinogenase shedaoenase | Q6T5L0 | 26416/6.7 | *Gloydius shedaoensis* | P | Serine protease |
| 42 | Snake venom serine protease serpentokallikrein-2 | Q9DG84 | 28320/6.58 | *Protobothrops mucrosquamatus* | T | Serine protease |
| 43 | Snaclec agkicetin-C subunit alpha | Q9DEA2 | 17798/8.83 | *Deinagkistrodon acutus* | P | C-type lectin |
| 44 | Snaclec agkisacutacin subunit B | Q8JIW1 | 16726/5.26 | *Deinagkistrodon acutus* | P | C-type lectin |
| 45 | Snaclec agglucetin subunit alpha-1 | Q8JIV9 | 17317/5.44 | *Deinagkistrodon acutus* | P | C-type lectin |
| 46 | Akitonin | Q90WL9 | 14658/4.87 | *Deinagkistrodon acutus* | T | C-type lectin |
| 47 | ACF 1/2 A-chain | Q8JIW0 | 17108/6.51 | *Deinagkistrodon acutus* | T | C-type lectin |
| 48 | Snaclec agglucetin subunit beta-2 | Q8AYA3 | 17234/8.1 | *Deinagkistrodon acutus* | P | C-type lectin |
| 49 | Snaclec agkicetin-C subunit beta | Q9DEA1 | 16688/5.66 | *Deinagkistrodon acutus* | P | C-type lectin |
| 50 | Snaclec anticoagulant protein subunit B | Q9DEF8 | 16997/5.25 | *Deinagkistrodon acutus* | P | C-type lectin |
| 51 | Snaclec agkisacutacin subunit A | Q9IAM1 | 17109/5.51 | *Deinagkistrodon acutus* | P | C-type lectin |
| 52 | Anticogulant protein subunit beta | I2GAE4 | 14554/5.02 | *Deinagkistrodon acutus* | T | C-type lectin |
| 53 | Snaclec agglucetin subunit alpha-2 | Q8AYA5 | 17977/4.92 | *Deinagkistrodon acutus* | P | C-type lectin |
| 54 | Snaclec clone 2100755 | Q8JIV8 | 17944/6.3 | *Deinagkistrodon acutus* | T | C-type lectin |
| 55 | Snaclec mamushigin subunit alpha | Q9YGG9 | 18333/8.66 | *Gloydius blomhoffii* | P | C-type lectin |

| No. | Protein name | Acc. no. | Theoretical mass/PI | Similar venom compound | | Function classified |
|---|---|---|---|---|---|---|
| | | | | Species | Evidence | |
| 56 | C-type lectin factor IX/X binding protein A subunit | T2HPA6 | 15512/8.45 | *Protobothrops flavoviridis* | T | C-type lectin |
| 57 | Snaclec stejaggregin-A subunit beta-1 | Q71RQ0 | 17019/5.51 | *Trimeresurus stejnegeri* | T | C-type lectin |
| 58 | Snake venom 5'-nucleotidase | B6EWW8 | 64433/8.65 | *Gloydius brevicaudus* | T | 5'-nucleotidase |
| 59 | 5'-nucleotidase | W8EFS0 | 45030/6.45 | *Macrovipera lebetina* | T | 5'-nucleotidase |
| 60 | Ecto-5'-nucleotidase 1 | U3FYP9 | 62982/8.93 | *Micrurus fulvius* | T | 5'-nucleotidase |
| 61 | 5'-nucleotidase | U3T7C6 | 55516/6.98 | *Ovophis okinavensis* | T | 5'-nucleotidase |
| 62 | 5' nucleotidase | T2HRS9 | 57090/8.27 | *Protobothrops flavoviridis* | T | 5'-nucleotidase |
| 63 | Venom phosphodiesterase 2 | J3SBP3 | 91751/8.39 | *Crotalus adamanteus* | P | Nuclease |
| 64 | Phosphodiesterase | U3TBJ5 | 100878/6.61 | *Ovophis okinavensis* | T | Nuclease |
| 65 | Acidic phospholipase A2 | Q7SID6 | 14032/4.68 | *Deinagkistrodon acutus* | P | phospholipase A2 |
| 66 | Basic phospholipase A2 homolog acutohaemolysin | O57385 | 15777/8.82 | *Deinagkistrodon acutus* | P | phospholipase A2 |
| 67 | Basic phospholipase A2 DAV-N6 | Q1ZY03 | 15853/8.62 | *Deinagkistrodon acutus* | P | phospholipase A2 |
| 68 | Acidic phospholipase A2 | P84651 | 13901/4.95 | *Lachesis stenophrys* | P | phospholipase A2 |
| 69 | Disintegrin accutin | P0DM77 | 5333/6.18 | *Deinagkistrodon acutus* | P | Disintegrin |
| 70 | Trimucrin | Q7T1S0 | 7876/6.42 | *Protobothrops mucrosquamatus* | T | Disintegrin |
| 71 | L-amino-acid oxidase | Q90W54 | 57091/6.53 | *Gloydius blomhoffii* | P | LAO |
| 72 | Aminopeptidase A | T2HQ95 | 109903/5.66 | *Protobothrops flavoviridis* | T | Aminopeptidase |

| No. | Protein name | Acc. no. | Theoretical mass/PI | Similar venom compound | | Function classified |
|---|---|---|---|---|---|---|
| | | | | Species | Evidence | |
| 73 | Helicopsin | P0DJG8 | 2620/4.78 | *Helicops angulatus* | P | Neurotoxin |
| 74 | Phospholipase B-like 1 | V8ND68 | 58279/8.1 | *Ophiophagus hannah* | Pre | other protein |
| 75 | Phospholipase B | T2HP68 | 64233/8.74 | *Protobothrops flavoviridis* | T | other protein |
| 76 | Uncharacterized protein | G1KH77 | 61267/7.51 | *Anolis carolinensis* | H | other protein |
| 77 | Uncharacterized protein | R4GAI6 | 48910/4.91 | *Anolis carolinensis* | H | other protein |
| 78 | Uncharacterized protein | G1KUG 5 | 50674/5.07 | *Anolis carolinensis* | H | other protein |
| 79 | Uncharacterized protein | R4GB02 | 66639/7.1 | *Anolis carolinensis* | H | other protein |
| 80 | Uncharacterized protein | H9GF03 | 76096/5.23 | *Anolis carolinensis* | Pre | other protein |
| 81 | Actin, alpha skeletal muscle | T1E7F6 | 42051/5.23 | *Crotalus horridus* | T | other protein |
| 82 | Venom nerve growth factor | Q3I5F4 | 27382/7.59 | *Oxyuranus scutellatus scutellatus* | P | other protein |
| 83 | Glutaminyl-peptide cyclotransferases | M9NCI9 | 42295/8.52 | *Trimeresurus gracilis* | T | other protein |
| 84 | Vascular non-inflammatory molecule 2 | V8N7Y3 | 97658/5.33 | *Ophiophagus hannah* | Pre | other protein |
| 85 | Cysteine-rich seceretory protein Da-CRPa | F2Q6G0 | 24743/5.96 | *Deinagkistrodon acutus* | T | other protein |
